# P-1142. Prevalence of Healthcare-Associated Infections: 2023 Point Prevalence Survey in 25 Tennessee Acute Care Hospitals

**DOI:** 10.1093/ofid/ofaf695.1336

**Published:** 2026-01-11

**Authors:** Casey Morrell, Dipen Patel, Melphine Harriott, Christopher Wilson

**Affiliations:** Tennessee Department of Health, Healthcare-Associated Infections and Antimicrobial Resistance, Nashville, Tennessee; HAI/AR, Tennessee Department of Health, Nashville, TN; TN Department of Health, Nashville, Tennessee; Tennessee Department of Health, Nashville, Tennessee

## Abstract

**Background:**

The Tennessee Department of Health (TDH), alongside the Centers for Disease Control and Prevention’s Emerging Infections Program, conducted point prevalence surveys in acute care hospitals within the state to evaluate healthcare-associated infections (HAIs). Surveys from 2011 and 2015 determined the prevalence of HAIs in Tennessee as 3.57% and 1.68% respectively. TDH repeated the survey in 2023 with preliminary state results presented here.

**Methods:**

TDH recruited 25 hospitals to participate, prioritizing hospitals that participated in 2011 or 2015 and a mix of hospital sizes. Hospital size was categorized by staffed beds with small being < 150 beds, medium 150–399, and large ≥400. Each hospital selected a survey day between May 1 and October 30, 2023. Data collected included patient demographics, clinical characteristics, medical device use, hospital locations, discharge outcome, and HAIs based on 2023 National Healthcare Safety Network (NHSN) definitions. Descriptive statistics were used, including *t* Tests, χ^2^ tests, and Fisher's exact tests, to summarize characteristics of study participants, comparing HAI cases with those patients not experiencing a HAI. Patients less than 2 years old were excluded from this analysis.

**Results:**

1,440 patients were included. 33 were assessed as experiencing an attributable HAI during their current admission (2.29%) (Table 1). Patients at large hospitals experienced HAIs at higher rates than medium and small hospitals (4.33% vs 2.28% vs 1.08%, p = 0.02). Patients with urinary catheters experienced a higher incidence of HAIs (4.30% vs 1.76%, p = 0.02), as did patients with central lines (7.35% vs 1.46%, p < 0.01). Patients with an HAI had a longer length of stay than those without (27.61 days vs 9.83 days, p < 0.01). Patients with an identified HAI had a higher rate of death as the admission outcome (12.12% vs 2.27%, p = 0.02).

**Conclusion:**

Higher incidence of HAIs in larger hospitals and among patients with medical devices, such as urinary catheters and central lines, highlights the need for targeted infection control measures. These findings reinforce earlier research on HAIs, emphasizing the need for ongoing and proactive efforts to combat these persistent threats, minimize HAIs, and safeguard patient safety.

**Disclosures:**

All Authors: No reported disclosures

